# TeaDiseaseNet: multi-scale self-attentive tea disease detection

**DOI:** 10.3389/fpls.2023.1257212

**Published:** 2023-10-11

**Authors:** Yange Sun, Fei Wu, Huaping Guo, Ran Li, Jianfeng Yao, Jianbo Shen

**Affiliations:** ^1^ School of Computer and Information Technology, Xinyang Normal University, Xinyang, China; ^2^ Research Center of Precision Sensing and Control, Institute of Automation, Chinese Academy of Sciences, Beijing, China; ^3^ Henan Key Laboratory of Tea Plant Biology, Xinyang Normal University, Xinyang, China; ^4^ Intelligent Equipment Research Center, Beijing Academy of Agriculture and Forestry Sciences, Beijing, China

**Keywords:** tea disease detection, deep learning, multi-scale feature, self-attention, convolutional neural networks

## Abstract

Accurate detection of tea diseases is essential for optimizing tea yield and quality, improving production, and minimizing economic losses. In this paper, we introduce TeaDiseaseNet, a novel disease detection method designed to address the challenges in tea disease detection, such as variability in disease scales and dense, obscuring disease patterns. TeaDiseaseNet utilizes a multi-scale self-attention mechanism to enhance disease detection performance. Specifically, it incorporates a CNN-based module for extracting features at multiple scales, effectively capturing localized information such as texture and edges. This approach enables a comprehensive representation of tea images. Additionally, a self-attention module captures global dependencies among pixels, facilitating effective interaction between global information and local features. Furthermore, we integrate a channel attention mechanism, which selectively weighs and combines the multi-scale features, eliminating redundant information and enabling precise localization and recognition of tea disease information across diverse scales and complex backgrounds. Extensive comparative experiments and ablation studies validate the effectiveness of the proposed method, demonstrating superior detection results in scenarios characterized by complex backgrounds and varying disease scales. The presented method provides valuable insights for intelligent tea disease diagnosis, with significant potential for improving tea disease management and production.

## 1 Introduction

As one of the traditional cash crops, tea holds significant economic and cultural value. However, the susceptibility of tea plants to diseases during their growth stages has a detrimental effect on both yield and quality, leading to significant economic losses for tea growers (Mukhopadhyay et al., 2021; Mahum et al., 2023; Sunil et al., 2023). Conventional manual techniques for detecting tea diseases are laborious, time-consuming, and dependent on the expertise of the testers, leading to inefficiency and high expenses (Drew, 2019; Abade et al., 2021). Additionally, the complex tea plantation environment, including elements like weeds, branches, and soil, along with factors like varying disease scales and densely shaded areas, pose challenges for accurately detecting of tea diseases. Therefore, there is an urgent need for research on rapid and precise methods for early detection of tea diseases. Implementing such methods would enable tea farmers to promptly implement control measures, prevent disease spread, protect the health of tea plantations, and promote the sustainable development of the tea industry (Debnath et al., 2021; Lanjewar and Panchbhai, 2023; Pandian et al., 2023).

Traditional machine learning models, such as support vector machines (SVM), decision trees, K-means, and random forests, require manual feature design specific to different disease types, making them susceptible to environmental factors and lacking generalization capabilities (Bhavsar et al., 2022; Zou et al., 2020; Steven, 2021; Yu et al., 2021; Bao et al., 2022; Prabu et al., 2022). Conversely, deep learning, particularly in object detection, exhibits potential in crop disease identification (Krisnandi et al., 2019; Ayan et al., 2020; Jiang et al., 2020; Tetila et al., 2020; Xiong et al., 2020; Hu et al., 2021b). However, existing models that solely consider local pixel relationships at short distances struggle to incorporate crucial global information in complex scenarios of tea disease detection, featuring varying disease scales and complex backgrounds, leading to limitations in detection accuracy (Li et al., 2021).

Convolutional Neural Networks (CNNs) have demonstrated remarkable success in automatically learning multi-level, high-order features from disease images, surpassing the limitations of traditional manual feature design methods (Abade et al., 2021; Akanksha et al., 2021; Dhaka et al., 2021; Latha et al., 2021; Lu et al., 2021; Wang et al., 2021; Yogeshwari and Thailambal, 2021; Ashwinkumar et al., 2022). They offer significant advantages in disease detection and have been extensively studied (Liu et al., 2022; Kirti and Rajpal, 2023; Kirti et al., 2023; Sudhesh et al., 2023; Tholkapiyan et al., 2023; Xu et al., 2023; Zhou et al., 2023). Depending on their network structure, CNN-based disease detection methods can be categorized as one-stage or two-stage detectors (Jiao et al., 2021; Lin et al., 2023). Regarding tea disease detection techniques, Qi et al. introduced TC-YOLO, a lightweight deep learning architecture based on YOLO that achieves high fusion capabilities (Qi et al., 2022). Alruwaili et al. improved the Faster R-CNN model for disease detection and achieved better recognition performance than other models (Alruwaili et al., 2022). By utilizing basic convolutional layer architectures, Lee et al. achieved an accuracy of 77.5% in detecting insect pests and diseases (Lee et al., 2020). Hu et al. introduced an algorithm that enhances image quality to improve detection accuracy (Hu et al., 2021a). Chen et al. developed LeafNet, a specialized CNN model for tea disease feature extraction (Chen et al., 2019). Xue et al. proposed YOLO-tea, a tea disease detection model based on YOLOv5 (Xue et al., 2023). However, CNNs overlook crucial global information among distant pixels, which impacts detection accuracy. Researchers are currently exploring methods to enhance the global modeling capabilities of CNNs in these scenarios. For instance, Hou et al. proposed an improved two-stage Faster R-CNN disease detection algorithm incorporating an attention mechanism in the network (Hou et al., 2023).

Attention mechanisms have emerged as highly successful approaches in disease detection tasks, aiming to emulate the remarkable capabilities of the human visual system in capturing vital information from complex scenes (Zheng et al., 2021; Hu et al., 2023). Spatial attention, channel attention, and self-attention are different attention mechanisms that enhance feature extraction and model performance (Carion et al., 2020; Guo et al., 2022). Several studies have employed attention mechanisms in disease detection models. For instance, Liu et al. proposed the spatial attention module (Liu et al., 2019), Wang et al. introduced both channel and spatial attention mechanisms (Wang et al., 2020), Zha et al. developed a lightweight network model based on a coordinate attention mechanism (Zha et al., 2021), Zhu et al. combined CNNs with Transformer architecture to establish (Zhu et al., 2022). Similarly, Lin et al. proposed a YOLO-based algorithm that employs a self-attentive mechanism to enhance the model’s ability to capture global information on tea diseases (Lin et al., 2023). Borhani et al. proposed combining CNNs with Transformer architecture to exploit the Transformer’s capability to establish dependencies between distant features and extract global disease features (Borhani et al., 2022). By incorporating attention mechanisms, researchers have made considerable progress in capturing essential information and enhancing the performance of disease detection models (Alirezazadeh et al., 2023; Yang et al., 2023).

Although the studies mentioned above have made progress in considering local disease information, it is crucial to emphasize the value of global information, especially the interaction between distant pixels (Sapna et al., 2023). Motivated by these challenges and research gaps, we introduce a novel network named Tea Disease Network (TeaDiseaseNet). Our proposed network integrates multi-scale feature representation with a self-attention mechanism to enhance performance in complex backgrounds and variable disease scales. The primary contributions can be summarized as follows:

(1) Introducing the Multi-scale Feature Extraction Module (MFEM), which utilizes multi-scale convolutional neural networks (CNNs) to capture comprehensive and localized multi-scale feature representations from disease images effectively. This module facilitates the extraction of comprehensive local spatial information.
(2) Devising the Scale Self-Attentive Module (SSAM) to address scale variations and complex backgrounds. The SSAM incorporates self-attention blocks to consolidate local and global information on tea disease images, facilitating effective interaction between global information and local features.
(3) Designing the Scale-aware Feature Fusion module (SFF) to achieve accurate and robust detection. The SFF enables feature fusion and the network to distinguish the relative importance of different input features. It enhances the accuracy and robustness of tea disease detection by facilitating multi-scale feature fusion.
(4) Conducting extensive comparative experiments and ablation studies on each module to demonstrate our proposed method’s superior performance and effectiveness. The results show significant improvements in various scenarios, surpassing most existing methods. These findings highlight the potential and effectiveness of our approach in enhancing the detection of tea diseases.

The structure of this paper is as follows. Section 2 focuses on the dataset utilized in this research and explains the enhanced modules integrated into TeaDiseaseNet. Section 3 covers the experimental setup, including equipment configuration, evaluation criteria, and experimental parameters. We present the results and analysis of the ablation experiments, visualization, and discussion. Finally, in Section 4, we present our conclusions and discuss potential avenues for future research.

## 2 Materials and methods

In this section, we outline the critical components of our proposed TeaDiseaseNet detection method. Our method involves two main aspects: collecting a comprehensive tea disease dataset and developing of an accurate disease detection framework. The dataset collection process includes acquiring disease images, annotating the dataset, and appropriately partitioning it. The detection model comprises three crucial functional modules: 1) The Multi-scale Feature Extraction Module (MFEM) extracts features from different scales to capture detailed information about tea diseases. 2) The Scale Self-Attention Module (SSAM) applies self-attention mechanisms to learn contextual dependencies within the extracted features. 3) The Scale-aware Feature Fusion (SFF) module fuses the multi-scale and self-attended features to generate a robust representation for disease detection. Collectively, these components contribute to the effectiveness and accuracy of our TeaDiseaseNet detection.

### 2.1 Tea disease dataset construction

#### 2.1.1 Disease images acquisition

The tea disease dataset utilized in the experiments was obtained from Professor Jiang Zhaohui’s research group at Anhui Agricultural University (Tholkapiyan et al., 2023). This dataset consists of 776 samples and covers a wide range of tea diseases, including tea exobasidium blight, tea red scab, tea cloud leaf blight, tea cake, tea red rust, and tea algae leaf spot. Each sample image in the dataset has a resolution of 906×600 pixels, ensuring a clear and detailed representation of the tea diseases.

Incorporating diverse tea diseases into the dataset enables comprehensive training and evaluation of the proposed detection model. By including samples from different tea diseases, the dataset offers a rich and representative collection of real-world scenarios encountered by tea growers.

**Figure 1:**
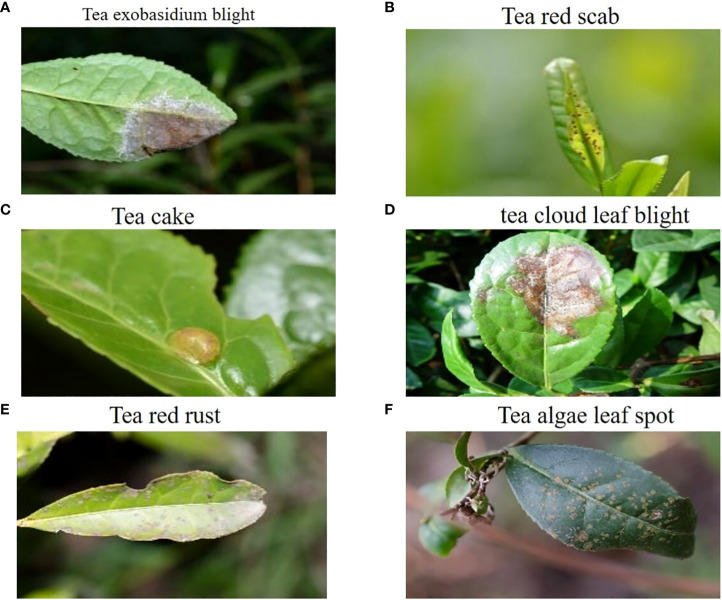
Representative Samples from the Tea Dataset.

**Figure 1** visualizes the dataset, displaying selected tea images that exemplify instances of the six tea diseases above. These images serve as valuable references for understanding each tea disease visual characteristics and distinguishing features. The annotated dataset ensures accurate labeling and facilitates the development of an effective convolutional neural network for tea disease detection.

By utilizing this meticulously collected and annotated dataset, we aim to construct a robust and reliable detection model capable of accurately identifying and classifying tea diseases. The dataset serves as a crucial foundation for our research, ensuring the validity and effectiveness of our proposed TeaDiseaseNet.

#### 2.1.2 Data labeling

To adapt the dataset for tea disease detection tasks, we enhanced the original samples by manually annotating the bounding boxes of the tea disease targets. This critical step involved meticulously labeling each sample image to indicate the precise location and extent of the tea disease instances. The annotation process was performed by a skilled professional using the labelimg image labeling tool, ensuring accuracy and consistency throughout the dataset.

By providing bounding box annotations, we enable the tea disease detection model to identify the presence of tea diseases localize and delineate the specific areas affected by the diseases. This level of detail enhances the model’s ability to provide valuable insights and facilitate targeted intervention strategies for tea growers.

The inclusion of bounding box annotations in the dataset enhances its suitability and efficacy for tea disease detection tasks. When used with our advanced TeaDiseaseNet algorithm, the annotated dataset enables accurate and precise detection of tea diseases.

#### 2.1.3 Data augmentation and division

To enhance the model’s generalization capability and improve its performance in real-world scenarios, data augmentation techniques were applied to augment the tea disease dataset, thereby expanding its size and diversity. Various methods introduced diversity and variability into the original images, including 90-degree clockwise and counterclockwise rotations, random rotation, noise addition, and exposure adjustments. As a result, a total of 7 640 augmented samples were generated, enriching the dataset and providing a more comprehensive range of training examples for the model.

The augmented dataset was subsequently divided into an 8:2 ratio for training and validation purposes. This division ensured a balanced distribution of data and enabled robust model evaluation. By training the model on a diverse augmented dataset and validating it on separate samples, we obtained more reliable and accurate results. The use of data augmentation techniques, along with the appropriate dataset division, enhances the model’s ability to accurately detect tea diseases, even when faced with previously unseen or challenging images.

#### 2.1.4 Characteristics of disease dataset

The dataset’s statistical analysis and ranking of scales revealed a significant range of sizes among the tea disease targets. Around 20% of the targets exhibited scales smaller than 0.0207, while 34% had scales larger than 0.345. This wide range of scales underlines the diverse nature of the dataset and emphasizes the challenge of accurately detecting diseases across various sizes. Understanding these scale variations is crucial for developing a robust detection model capable of effectively identifying tea diseases, regardless of their size. Our goal is to enhance the performance and reliability of the model in detecting tea diseases by addressing the scale variations.

### 2.2 The architecture of TeaDiseaseNet

**Figure 2:**
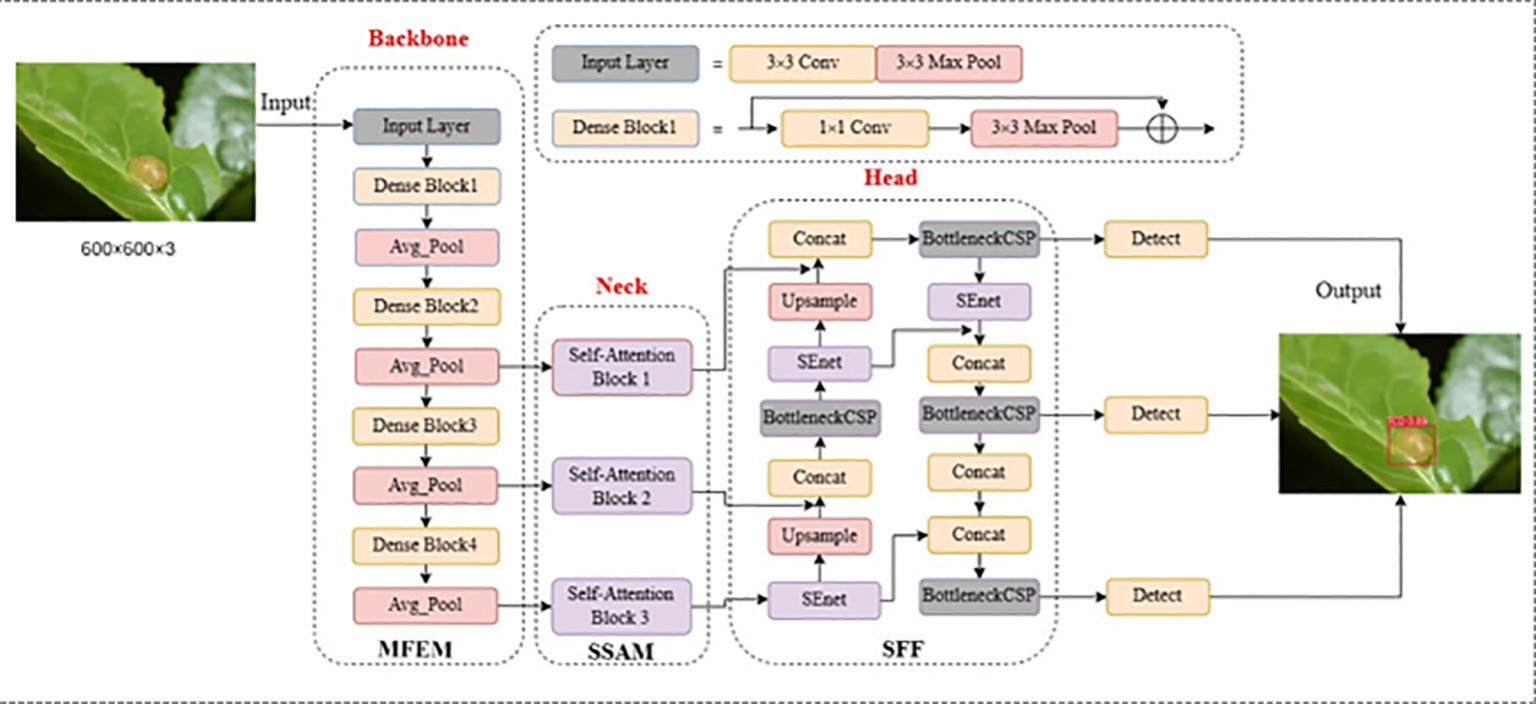
The framework of TeaDiseaseNet.

To address the challenges posed by variable scales of tea pests and dense, obscuring diseases, this paper presents a novel fused multi-scale self-attentive tea disease detection network based on improving YOLOv5 (Jocher et al., 2022). The YOLOv5 framework is well-known for its remarkable object detection capabilities and high efficiency. In our proposed model, we have harnessed the advantages of YOLOv5 by incorporating multi-scale convolution and multi-scale self-attention mechanisms to effectively capture both local and global features in tea disease images. **Figure 2** illustrates the network structure of our model, which comprises three key modules: the Multi-scale Feature Extraction Module (MFEM), the Scale-Self-Attention Module (SSAM), and the Scale-aware Feature Fusion (SFF). These modules synergistically work to achieve accurate and robust tea disease detection. Our approach involves the following steps:

**Step 1: Multi-scale feature extraction**
  We utilize the multi-scale convolutional blocks of the MFEM as a backbone network to extract features from tea images. This allows us to capture feature information of tea diseases at different scales and local levels.
**Step 2: Scale self-attentive mechanism**
  We feed the multi-scale feature maps into the SSAM simultaneously to enable the interaction of global and local information. This mechanism dynamically adjusts the weights of each scale feature, improving the model’s ability to capture the characteristics of tea diseases.
**Step 3: Scale-aware feature fusion**
  We incorporate a channel attention mechanism to perform a weighted fusion of features at different scales in tea leaf images. This mechanism efficiently integrates characteristic information of tea diseases across a wide range of scales, enhancing the precision of disease localization and recognition.
**Step 4: Prediction**
  The prediction module utilizes the previously extracted feature information to efficiently localize and identify tea disease features in complex contexts and at varying scales.

By following these steps, our approach aims to effectively extract and integrate features to accurately detect and recognize tea diseases.

### 2.3 Multi-scale feature extraction module

Traditional image feature extraction methods often focus on either local or global information, limiting their ability to comprehensively capture the diversity and complexity of images. In recent years, deep learning-based approaches, particularly Vision Transformers (ViT) (Khan et al., 2022), have become the dominant method for image feature extraction. ViT segments images into patches or tokens and employs self-attentive mechanisms to extract parameterized visual representations. However, these methods are constrained by fixed-scale token sequences, which restrict their ability to capture feature structures across different scales. This limitation poses a challenge in tea disease detection due to scale variations. Moreover, self-attentive mechanisms prioritize global information, disregarding important local feature details and blurring the distinction between intricate backgrounds and foregrounds in tea disease images. Consequently, their applicability in disease feature extraction tasks is limited.

**Figure 3:**
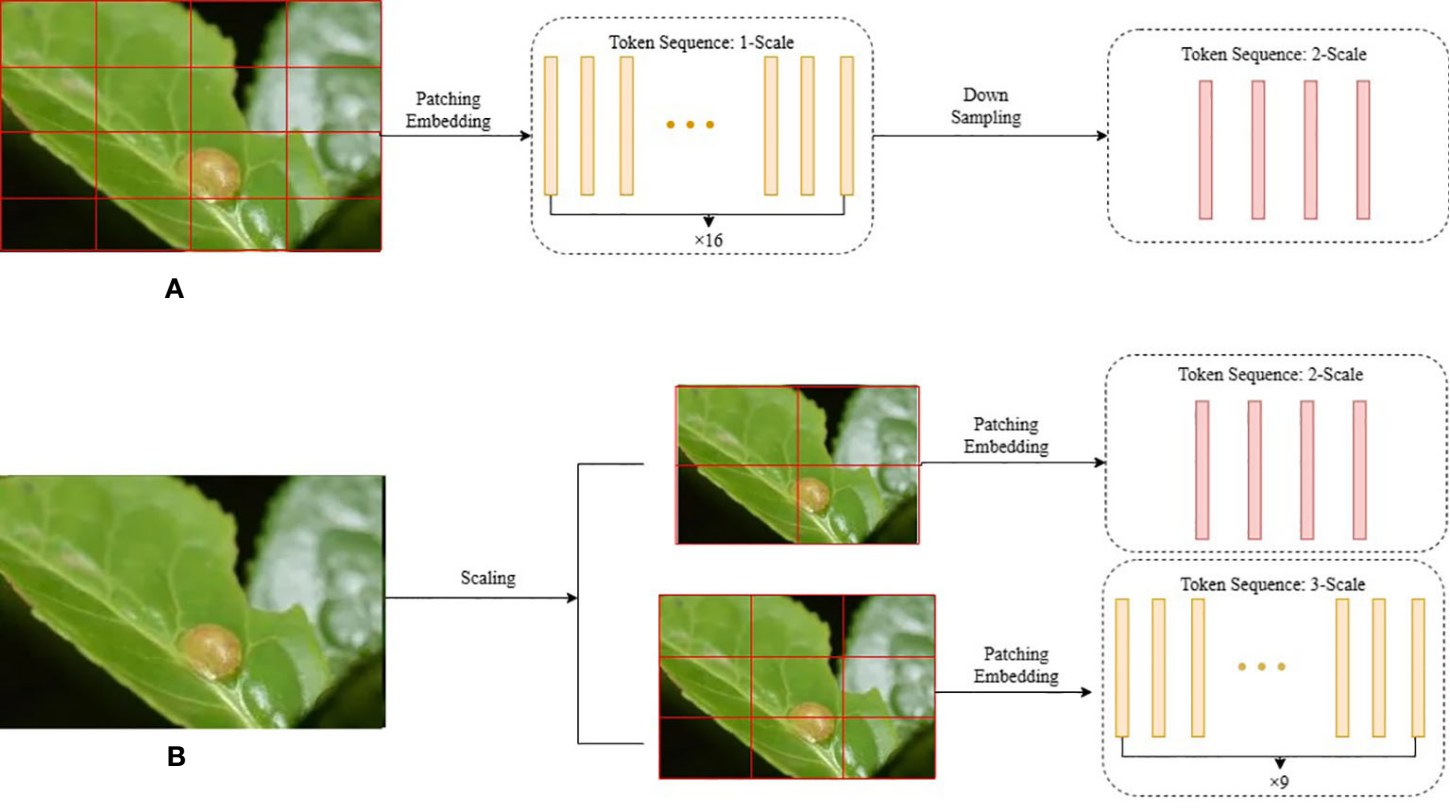
Two Ideas for Multi-scale Structures in Tea Disease Detection. **(A)** Constructing serial multi-scale token sequences. **(B)** Constructing parallel multi-scale sequences.

To address these challenges, we propose two solutions. The first solution, illustrated in **Figure 3A**, involves constructing serial multi-scale token sequences by up/down sampling and expanding/reducing token sequences within the self-attentive mechanism module. The second solution, depicted in **Figure 3B**, consists of constructing parallel multi-scale token sequences wherein images of different scales are simultaneously fed into the self-attentive mechanism module. This approach leverages multi-headed self-attention to capture global contextual information across diverse scales. Compared to the first solution, the second approach provides a simpler implementation. Building on these observations, we propose a parallel multi-scale tea disease feature extraction module to address the limitations of limited local feature representation and a single scale.

As illustrated in **Figure 2**, our proposed method employs four Dense blocks derived from DenseNet (Roy and Bhaduri, 2022) to extract both the multi-scale structure and local features of tea disease images. The tea disease image passes through the input layer, further progressing into the dense block, and finally undergoing average pooling. The shallow convolutional layers in this module aim to capture intricate features like edges and contours, while the deeper convolutional layers encode comprehensive semantic information. Each level of the Dense block includes down-sampling operations, gradually reducing the resolution of the disease images. We generate a multi-scale feature map by preserving the outputs of the last three levels of Dense blocks. By employing the scaled feature map sequence obtained from the CNN as input for the self-attentive mechanism module, the length of the token sequence is indirectly adjusted. This modification enables each token to represent a larger region in the original image, encompassing a broader range of spatially localized information.

In summary, the MFEM module retrieves multi-scale features, allowing the model to capture information at different levels of detail. This capability is advantageous for tea disease detection tasks as it effectively handles disease size, location, and complex backgrounds variations. The refined multi-scale features enhance the reliability and accuracy of the tea disease detection model.

### 2.4 Scale self-attention module

The SSAM enables the interaction and fusion of feature maps at various scales using the self-attention mechanism. This allows the tea disease detection model to effectively capture both global and local information in disease images. More specifically, the self-attentive block within the SSAM module takes in multi-scale feature maps as inputs, with each scale’s feature maps obtained through convolution. By enhancing information fusion and interaction, this module significantly improves the model’s performance and accuracy across various scales. The self-attention operation in each head of the multi-head attention mechanism is computed based on Equation (1).


(1) $$A_{Attention}\left(Q,K,V\right)=S_{softMax}\left(\frac{QK^{T}}{\sqrt{d}}+B\right)V$$

where *Q*, *K*, and *V* represent the query, key, and value matrices, respectively. The vector dimension is denoted as *d*, and *B* signifies the bias matrix. The output is obtained by applying the softmax activation function *S*_softMax_ for multi-classification.

**Figure 4:**
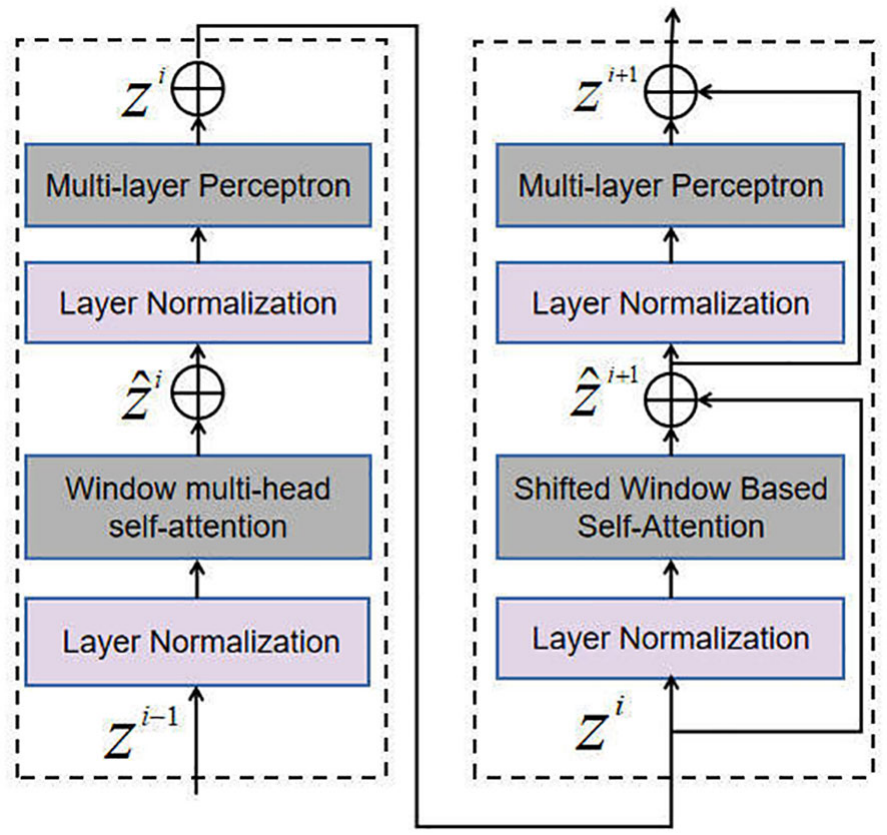
Self-Attention Block.


In particular, the Self-Attention Block within the SSAM takes multi-scale feature maps as input. Each scale is obtained through a convolution operation. The configuration of the Self-Attention Block, illustrated in **Figure 4**, includes a Multi-head Self-Attention (MSA) module that employs a window-based approach and a 2-layer Multi-layer Perceptron (MLP) module. Layer Normalization (LN) layers are incorporated before each MSA and MLP module, and residual connections are employed after each module. This arrangement facilitates the calculation of output features, as shown in Equation (2).

(2) $$\left\{ \begin{array}{l} {\hat{z}}^{i}=F_{W-MSA}\left(F_{LN}\left(z^{i-1}\right)\right)+z^{i-1}\\ z^{i}=F_{MLP}\left(F_{LN}\left({\hat{z}}^{i}\right)\right)+{\hat{z}}^{i}\\ {\hat{z}}^{i+1}=F_{SW-MSA}\left(F_{LN}\left(z^{i}\right)\right)+z^{i}\\ z^{i+1}=F_{MLP}\left(F_{LN}\left({\hat{z}}^{i+1}\right)\right)+{\hat{z}}^{i+1} \end{array}\right.$$

where W-MSA represents the window multi-head self-attention, while SW-MSA denotes the shifted window multi-head self-attention. The variables ${\hat{z}}^{i}$ and $z^{i}$ correspond to the output features of the (S)W-MSA and MLP modules of the *i*-th block, respectively. The W-MSA module, SW-MSA module, MLP module and LN module features are denoted as F_W-MSA_, F_SW-MSA_, F_MLP_, F_LN_, respectively.

### 2.5 Scale-aware feature fusion

The SFF module efficiently combines features from multiple scales, resolving discrepancies and improving model performance. In tea disease detection tasks, it is crucial to efficiently process information from multiple scales. This module is specifically designed to address discrepancies and inconsistencies in multi-scale features. We leverage a channel focus mechanism to enhance the model’s performance by incorporating spatial and channel features in the input data. This allows the model to accurately discern and differentiate between objects or features, improving object detection accuracy.

The channel attention mechanism enhances the inter-channel information importance in a convolutional neural network. It compresses the features of each channel by integrating their spatial information and computes them using global average pooling, as defined below:


(3) $$z=F_{sq}=\frac{1}{H\times W}\sum_{i=1}^{H}\sum_{i=1}^{W}X\left(i,j\right)$$

where *z* denotes the compressed feature vector, *H* and *W* denote the feature map size of feature *X*. A learnable parameter *w* captures the correlation between feature channels. To improve computational efficiency, the number of channels is reduced using the following approach:


(4) $$s=F_{ex}\left(z,w\right)=\partial \left(g\left(z,w\right)\right)=\left(w_{2}\delta \left(w_{1}z\right)\right)$$

where the adaptive weight of each channel is represented by *s*, and *δ* represents the ReLU activation function, while σ represents the Sigmoid activation function. Combining the channel adaptive weight *s* with the original feature *z* and assigning a new adaptive weight to each existing channel, the rescaled feature is obtained using Equation (5).


(5) $$Xc=F_{scale}\left(X,s\right)=X\cdot s$$

**Figure 5:**
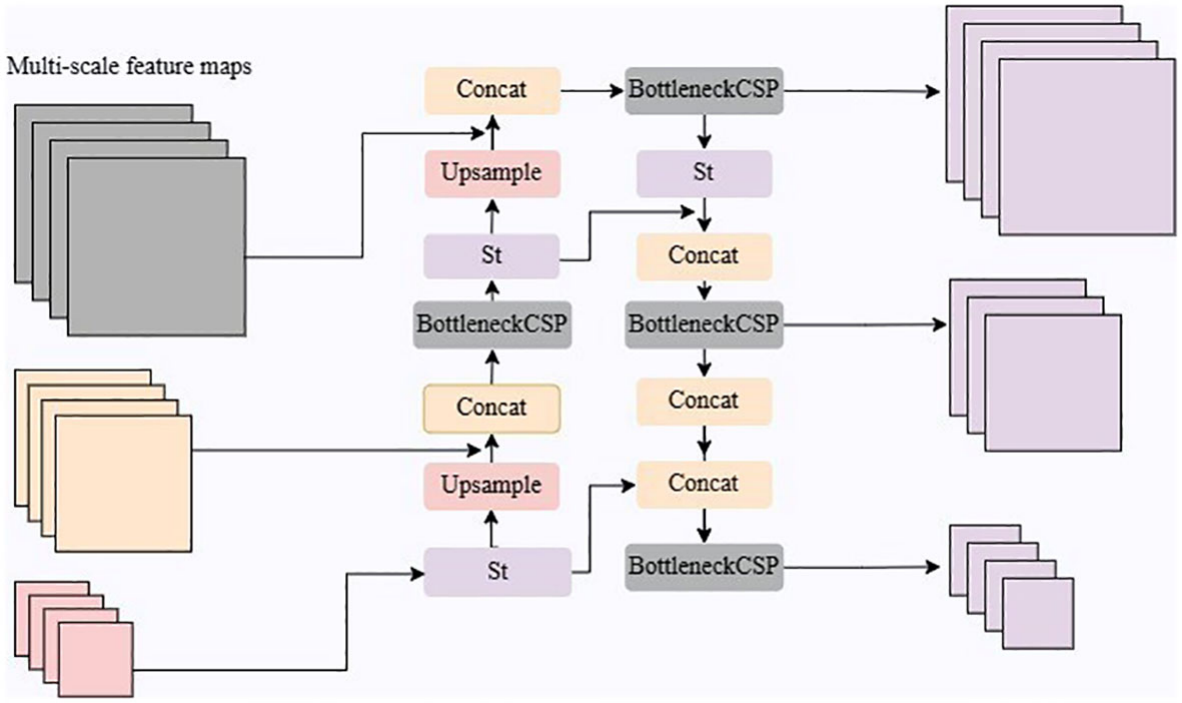
The multi-scale feature fusion module.

As shown in **Figure 5**, the SFF consists of Upsample, Concat, Bottleneck CSP, and *S_t_* module operations. The BottleneckCSP module performs a convolution operation on the fused features to further extract feature information, and the St module introduces a channel attention mechanism to weigh the multi-scale features for fusion and eliminate redundant information.

The *S_t_* module utilizes global average pooling to compute feature compression values for each channel and learns parameters to model the correlation between channels, resulting in adaptive weights. These weights are applied to rescaled original features, achieving adaptive feature weighting and improving feature representation. Through the combined operations of Upsample, Concat, BottleneckCSP, and *S_t_*, the feature fusion network enables the interaction and fusion of multi-scale information, enhancing the model’s performance. This addresses inconsistencies and discrepancies between multi-scale features, improving accuracy and robustness in tea disease detection tasks.

In general, the primary objective of the SFF module is to integrate global and local information from multiple scales, enabling the generation of precise density maps to effectively capture the spatial distribution of diseases.

### 2.6 Prediction module

The prediction module is responsible for locating and identifying tea disease information at various scales. It achieves efficient prediction by utilizing the feature fusion network’s output and employing parallel branches. The incorporation of IoU branches further enhances the accuracy of the prediction results.

The prediction module comprises 1×1 convolutional layers and parallel branches. Each branch contains a Conv Block that comprises two 3×3 convolutions. The topmost Conv Block is dedicated to the classification task, while the bottommost Conv Block focuses on the regression task. An additional IoU branch is introduced to the module to enhance the accuracy of the predictions.

### 2.7 Loss functions

The tea disease detection model utilizes three essential loss functions: localization loss $L_{loc}$ , classification loss $L_{cls}$ , and confidence loss $L_{conf}$ , as depicted in Equation (6).


(6) $$L=L_{loc}+L_{cls}+L_{conf}$$

These components enhance the model’s accuracy regarding object localization and category identification. The localization loss minimizes bounding box coordinate discrepancies, while the classification loss reduces errors in tea disease classification. Finally, the confidence loss enhances the model’s precise detection and classification estimation. The model can optimize its performance by incorporating these loss functions and achieve more accurate and reliable tea disease detection results.

The final localization loss $L_{loc}$ is computed according to Equation (7).


(7) $$L_{loc}=\sum_{i=1}^{s^{2}}\sum_{j=0}^{B}I_{i,j}^{obj}\left(1-CIoU\right)$$

The Complete Intersection over Union (CIoU) loss is a regression loss function considering bounding boxes’ overlapping area, center distance, and aspect ratio consistency. When incorporated into the model, it provides a more accurate measure of the bounding box regression error, leading to improved accuracy and localization performance in tea disease detection.


(8) $$CIoU=IoU-\frac{\rho^{2}\left(b,b^{gt}\right)}{c^{2}}-\alpha \upsilon$$

$$\nu =\frac{4}{\pi^{2}}{\left(\arctan\frac{w^{gt}}{h^{gt}}-\arctan\frac{w}{h}\right)}^{2}$$

$$\alpha =\frac{\upsilon }{\left(1-IoU\right)+\upsilon }$$

where *c* represents the diagonal distance between the prediction frame and the minimum enclosing area of the ground truth frame, *ρ* denotes the Euclidean distance function, while *b* and $b^{gt}$ correspond to the centroids of the prediction frame and the actual frame, respectively. The variable ν indicates the similarity of the aspect ratio, and *α* is the weighting factor. Additionally, *w* and *h* denote the width and height of the prediction frame, respectively.

A binary cross-entropy loss function is used for the classification loss $L_{loc}$ , according to Equation (9).


(9) $$L_{cls}=-\sum_{i=1}^{s^{2}}\sum_{j=1}^{B}\sum_{c\in classes}I_{i,j}^{obj}\left[{\hat{p}}_{i}^{j}\log\left(p_{i}^{j}\right)+\left(1-{\hat{p}}_{i}^{j}\right)\log\left(1-p_{i}^{j}\right)\right]$$

where *S*, *B* and $I_{i,j}^{obj}$ have the same meaning as in the context, *c* is the currently identified category and classes are all the classes to be detected, $p_{i}^{j}$ and ${\hat{p}}_{i}^{j}$ are the predicted and true probabilities that the target in the *i*-th grid, *j*-th anchor box belongs to class *c*, respectively. The confidence loss $L_{conf}$ is computed according to Equation (10).


(10) $$\begin{array}{l} L_{conf}=-\sum_{i=0}^{s^{2}}\sum_{j=0}^{B}I_{i,j}^{obj}\left[{\hat{C}}_{i}^{j}\log\left(C_{i}^{j}\right)+\left(1-{\hat{C}}_{i}^{j}\right)\log\left(1-C_{i}^{j}\right)\right]\\ -\lambda_{noobj}\sum_{i=1}^{s^{2}}\sum_{j=0}^{B}I_{i,j}^{noobj}\left[{\hat{C}}_{i}^{j}\log\left(C_{i}^{j}\right)+\left(1-{\hat{C}}_{i}^{j}\right)\log\left(1-C_{i}^{j}\right)\right] \end{array}$$

where, $I_{i,j}^{noobj}$ denotes the *i*-th grid, whether the *j*-th anchor box does not have a target, no target is 1, otherwise is 0; $\lambda_{noobj}$ is a constant coefficient, taken as 0.5, to balance the effect of positive and negative samples on the loss function; $C_{i}^{j}$ and ${\hat{C}}_{i}^{j}$ are the confidence levels of the prediction and truth boxes respectively.

## 3 Experimental results and analysis

The experiments were conducted using Python programming language and the PyTorch deep learning framework (version 1.7.0). Taking advantage of the server’s configuration, which included two RTX 3090 GPUs, the training process efficiently utilized parallel processing. The Adam optimizer was employed to optimize the training process. A batch size of 8 was selected, striking a balance between computational efficiency and model convergence. To ensure comprehensive learning and convergence, the models were trained for 300 epochs. This experimental setup effectively maximized computational resources, enabling accurate and reliable model training.

### 3.1 Performance comparisons

In this paper, we evaluate the performance of disease detection models using the mean Average Precision (mAP), Precision, and Recall as metrics. The mAP is calculated by summing the Average Precision values for all categories and dividing it by the total number of categories, as shown in Equation (11).


(11) $$AP=\int_{0}^{1}p(r)dr$$

$$mAP=\frac{1}{n}\sum_{i=1}^{n}AP_{i}$$

where *n* represents the class number, *AP_i_* denotes the Average Precision values for each category. This formulation enables a comprehensive and concise evaluation of the model’s overall detection accuracy, capturing its performance across diverse disease categories.

Precision provides valuable insights into the model’s capability to accurately identify and classify target frames. It quantifies the ratio of correctly identified frames to the total predicted frames, providing a measure of the model’s precision and accuracy in target detection. Equation (12) represents the mathematical expression of Precision.


(12) $$P=\frac{TP}{TP+FP}$$

Recall is defined as the ratio of correctly detected target frames to the total number of target frames in the dataset, assessing the model’s ability to identify all instances of the target without missing any. Equation (13) represents the mathematical expression for Recall.


(13) $$R=\frac{TP}{TP+FN}$$

This study evaluates the performance of TeaDiseaseNet by comparing and analyzing its detection and identification results with various classical CNN models, including SSD (Liu et al., 2016), Faster R-CNN (Ren et al., 2015) YOLOv3 (Redmon and Farhadi, 2018), YOLOv4s (Bochkovskiy et al., 2020), YOLOv5s (Jocher et al., 2022), YOLO-Tea (Xue et al., 2023), and AX-RetinaNet (Bao et al., 2022). **Table 1** presents these networks’ detection and recognition experiments’ precision, recall, and mean Average Precision (mAP) values. The results demonstrates the outstanding detection accuracy of TeaDiseaseNet. TeaDiseaseNet achieves superior detection accuracy compared to models that employ model scaling, such as YOLOv4s and YOLOv5s. This remarkable performance can be attributed to the utilization of DenseNet, which incorporates dense connectivity in the network, enhancing feature reuse and gradient flow. Moreover, TeaDiseaseNet employs effective techniques for multi-scale feature extraction and fusion.

**Table 1:** The Comparison of different networks.

Network	Year	Precision (%)	Recall (%)	mAP (%)
SSD	2016	86.5	89.1	88.4
Faster R-CNN	2015	91.5	87.3	92.2
YOLOv3	2018	94.2	84.6	90.3
YOLOv4s	2020	90.7	85.9	88.7
YOLOv5s	2020	92.3	86.5	89.4
AX-RetinaNet	2022	96.8	94	93.8
YOLO-Tea	2023	–	–	79.3
TeaDiseaseNet	2023	95.3	97.1	93.5

It is worth noting that the YOLOv3 algorithm exhibits higher detection accuracy than YOLOv4s and YOLOv5s, potentially because of its shallower depth and smaller feature map width. The detection accuracy of Faster R-CNN is higher than that of YOLOv3 by 1.9%. This performance difference arises because Faster R-CNN is a two-stage target detection algorithm. It generates candidate regions using a region proposal network and selects the best candidate regions using a region classification network. In contrast, YOLOv3 is a one-stage target detection algorithm that predicts object locations and classes across the entire image by taking the entire image as input. Despite requiring more computational resources, Faster R-CNN delivers higher accuracy and fewer false positives compared to YOLOv3. Furthermore, TeaDiseaseNet demonstrates a slightly higher average accuracy compared to the SSD algorithm.

In conclusion, this paper presents a significant advancement in disease detection by employing CNN characteristics and incorporating a self-attentiveness mechanism. TeaDiseaseNet utilizes CNN to extract multi-scale feature maps that encompass abundant spatial information at various levels of detail. Inspired by human visual mechanisms, this design enhances the model’s capability to effectively handle complex backgrounds and scale variations in disease images. The incorporation of the attention mechanism empowers TeaDiseaseNet to automatically select and prioritize the most relevant features within an image, significantly enhancing disease detection accuracy.

**Figure 6:**
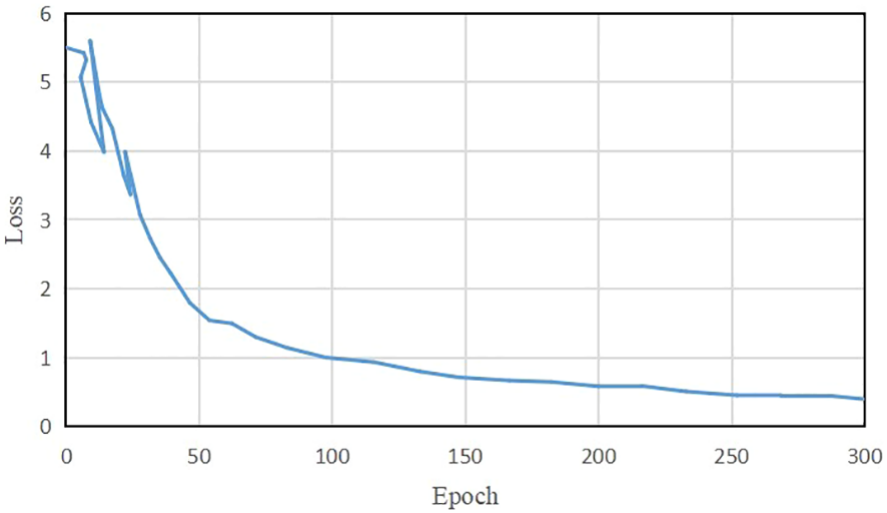
The Loss changing graph of TeaDiseaseNet.

**Figure 6** illustrates the average loss value curve of TeaDiseaseNet during training iterations. The plot demonstrates that the loss value stabilizes around 0.39 after approximately 255 iterations. The slight fluctuations observed in the loss value after convergence can be attributed to the inherent complexity and variability of the training data. The results indicate that TeaDiseaseNet has successfully learned and adapted to the training data, as evidenced by the convergence of the parameters and satisfactory performance.

### 3.2 Evaluation of TeaDiseaseNet

The effectiveness of the proposed TeaDiseaseNet algorithm was evaluated using the provided dataset. **Table 2** presents the accuracy of the model in recognizing each tea disease. The results highlight the significant advantages of the algorithm for tea disease detection. The algorithm achieved high accuracy rates of 92.1% and recall rates of 92.9% for tea round red star disease, with an average accuracy rate of 94.5%. These findings indicate accurate identification and significant improvements in detecting this particular disease. Tea webcake disease exhibits slightly lower accuracy and recall rates of 89.2% and 85.8%, respectively. However, the algorithm achieves an average accuracy rate of 95.4%, surpassing the individual accuracy and recall values. This demonstrates the algorithm’s capability to overcome challenges related to small spot areas. The algorithm also performs remarkably well in detecting other tea diseases, including tea algae spots, tea cake disease, tea cloudy leaf blight, and tea red rust algae disease. These diseases exhibit high values across all evaluation metrics. The results demonstrate significant innovations and benefits in multi-scale tea disease detection. The algorithm achieves high recognition accuracy for large-scale tea redscab and small-scale tea exobasidium blight.

**Table 2:** Performance in detecting different tea diseases.

Tea Disease	P (%)	R (%)	mAP (%)
Tea exobasidium blight	89.2	85.8	95.4
Tea red scab	92.1	92.9	94.5
Tea algae leaf spot	94.9	88.8	93.5
Tea cake	90.0	91.4	94.7
Tea cloud leaf blight	88.5	89.5	92.0
Tea red rust	85.4	87.7	90.9

The algorithm achieves high recognition accuracy for large-scale tea redscab and small-scale tea exobasidium blight. The performance evaluation of each network was conducted based on metrics such as accuracy, recall, and average accuracy, and the results are presented in **Table 3**. The results revealed that the DenseNet model, serving as the backbone network, performed the best in accuracy, recall, and average accuracy. The algorithm achieves high recognition accuracy for both large-scale tea redscab and small-scale tea exobasidium blight. DenseNet exhibits strong resistance to overfitting, making it particularly suitable for scenarios with limited training data. A notable characteristic of DenseNet is its utilization of feature reuse through feature concatenation across channels. This enables DenseNet to achieve superior performance compared to ResNet-101, while utilizing fewer parameters and incurring lower computational cost. In contrast, Darknet53 is a lightweight convolutional neural network, however, it proves to be challenging to train. DenseNet performs admirably in the tea disease detection task. As a result, this paper selects DenseNet as the underlying network structure for the proposed algorithm. The algorithm effectively resolves the scaling issue by establishing a multi-scale feature representation and enhances overall performance. In summary, the algorithm proposed in this study demonstrates improved accuracy compared to other models, thereby representing significant progress in the field of tea disease detection.

**Table 3:** Performance comparison of different backbone networks.

Backbone	P (%)	R (%)	mAP (%)
DenseNet	95.3	97.1	93.5
Resnet-101	91.3	90.2	92.8
Darknet53	91.8	90.5	93.2

### 3.3 Ablation studies

To validate the effectiveness of the proposed network model, incremental ablation experiments were conducted. Each network module was incrementally incorporated based on the DenseNet backbone architecture. This approach allowed for a comprehensive evaluation of each module’s contribution to the overall performance. This step-by-step approach aimed to enable a comprehensive evaluation of the individual contribution of each module to the overall performance.

The results of the ablation experiments conducted for each module are presented in **Table 4**. Including of the MFEM+SSAM module results in a substantial performance improvement, with a 2.2% increase in mAP compared to using the MFEM module alone. This improvement can be attributed to utilizing the multi-head self-attention mechanism within the MFEM+SSAM module. This mechanism captures global contextual information from multi-scale feature maps and facilitates the interaction between global and local information. Assigning weights to features, such as spot color and leaf edge, enhances the detection accuracy. Furthermore, the new scale-aware feature fusion (SFF) module adopts a channel attention mechanism to fuse features of different scales. It focuses on the feature channels containing discriminative information and assigns a higher weight distribution to them, effectively improving the detection performance (Chen et al., 2020). The SFF module effectively fuses information from tea disease features of various scales, resulting in improved accuracy of localization and identification. The introduction of the SFF module enhances the mAP by 0.7%, indicating its contribution to improved detection accuracy of the network.

**Table 4:** Results of ablation experiments.

Backbone (MFEM)	SSAM	SFF	mAP (%)
√			90.6
√	√		92.8
√	√	√	93.5

### 3.4 Visualization and discussion

**Figure 7:**
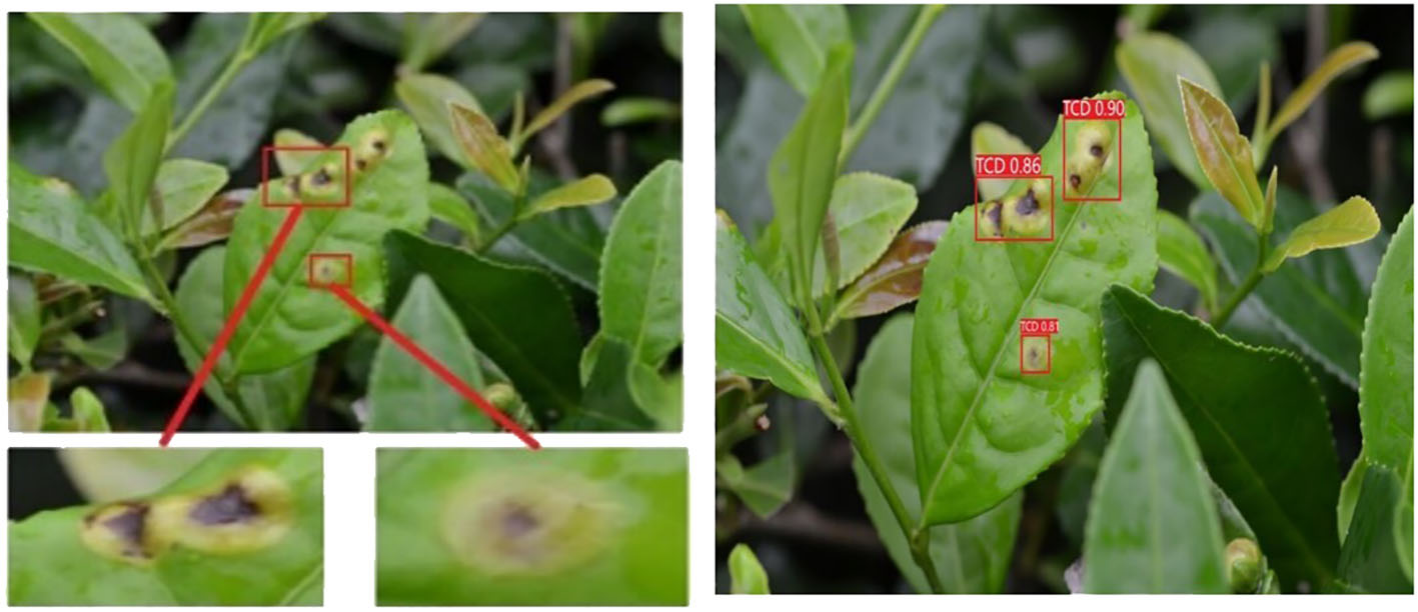
Disease scenarios with continuous scale changes.

**Figure 8:**
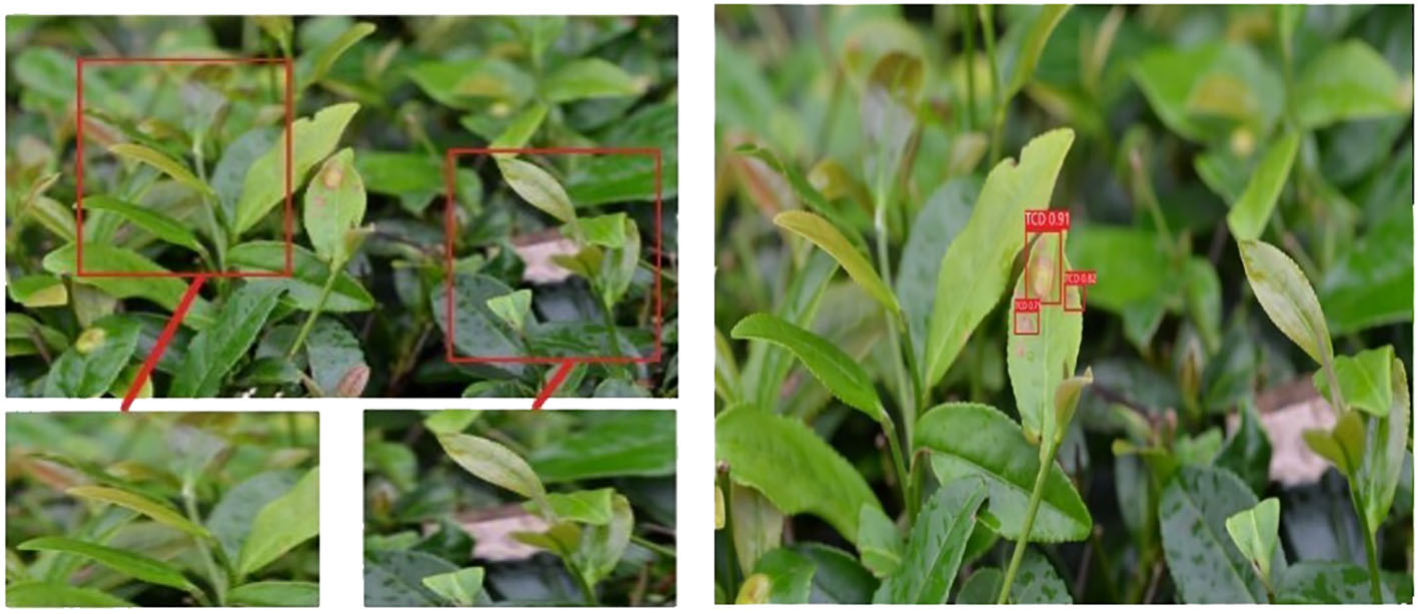
Disease scenes with complex backgrounds.

Representative disease images were selected to showcase the exceptional performance of the proposed model in effectively addressing challenges posed by continuous scale variations and complex backgrounds. This visualization demonstrates the model’s ability to detect diseases accurately. **Figures 7** and **8** present the original disease images on the left and the model’s detection results on the right. Rectangular boxes indicate the identified disease types and their corresponding confidence levels.

**Figure 7** demonstrates the model’s ability to effectively identify diseases with varying scales and address disease scenarios characterized by continuous scale variations. This showcases the algorithm’s capacity to extract rich global contextual information at multiple scales and accurately detect scale variations by comparing global and local information. **Figure 8** highlights the model’s effectiveness in eliminating complex background interferences, such as branches and fallen leaves in disease scenes. This can be attributed to the feature extraction and fusion networks, which enable the proposed method to accurately detect disease areas within complex scenes by capturing dependencies between input feature scales.

**Figure 9:**
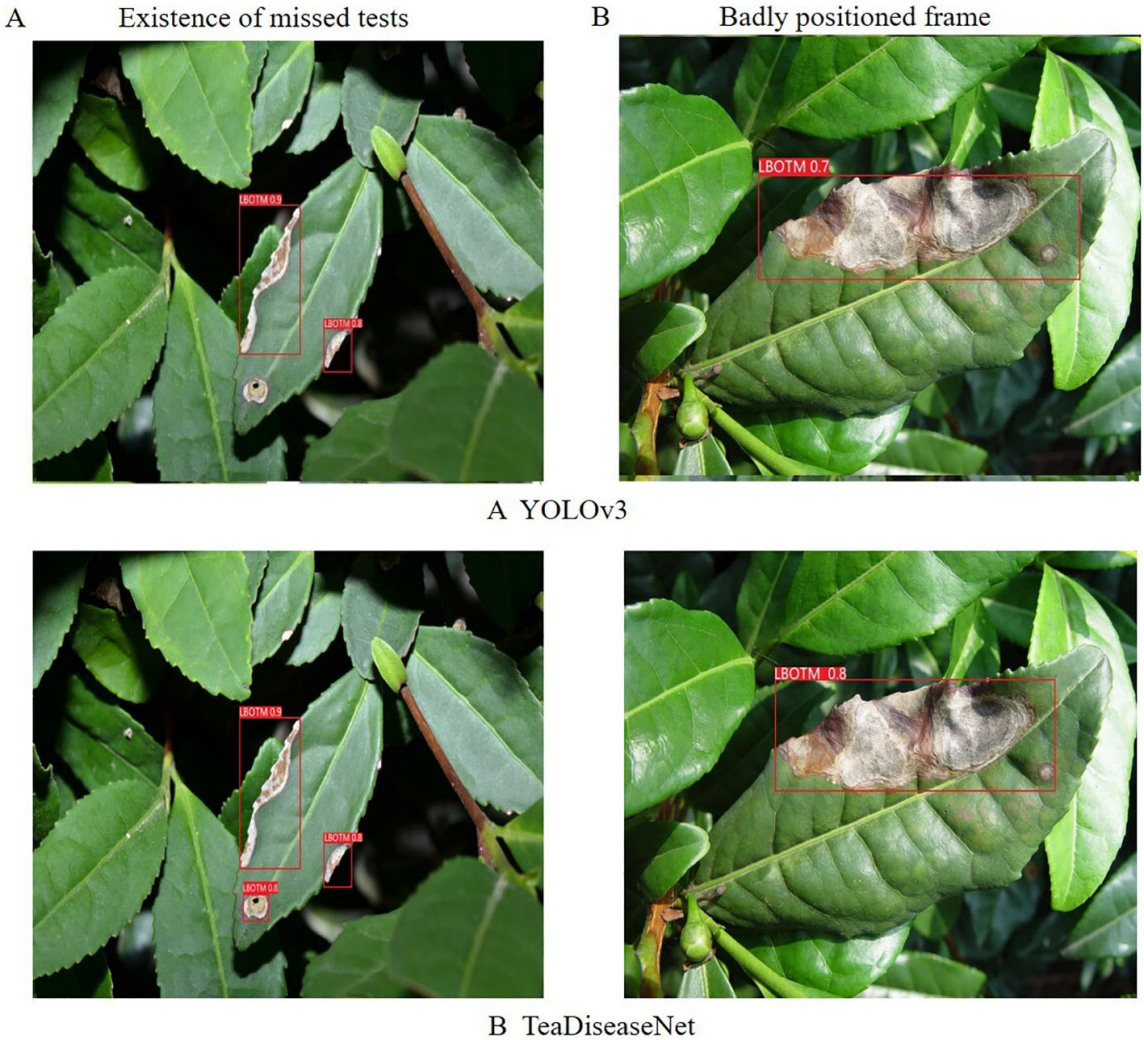
The results of algorithm **(A)** YOLOv3 and algorithm **(B)** TeaDiseaNet, in the existence of missed tests and badly positioned frame scenarios, respectively.

Additionally, a comparison was conducted between the YOLOv3 and TeaDiseaseNet models using images of tea leaf diseases, as depicted in **Figure 9**. The YOLOv3 model exhibited missed detections and inaccurate annotation box positions, whereas TeaDiseaseNet accurately detected and confidently annotated the diseases. The superior performance of TeaDiseaseNet can be attributed to its multi-scale self-attention mechanism, which enhances the acquisition of semantic and location information in the images. This results in improved feature extraction and detection accuracy.

The results demonstrate that TeaDiseaseNet outperforms YOLOv3 in terms of detection accuracy and robustness, owing to its enhanced feature extraction capabilities and multi-scale self-attention mechanism.

## 4 Conclusion

This paper introduces TeaDiseaseNet, a novel tea disease detection model that effectively addresses challenges posed by complex backgrounds and variable scales. By incorporating a multi-scale self-attentive mechanism, TeaDiseaseNet enables effective interactions between global and local features across multiple scales. This mitigates the impact of scale variations and complex backgrounds on detection accuracy. Experimental results demonstrate that TeaDiseaseNet surpasses state-of-the-art methods, exhibiting exceptional accuracy and robustness in scale variations and background interference scenarios. These findings provide valuable insights for intelligent tea disease diagnosis, supporting tea farmers with accurate detection capabilities and enabling timely control measures to protect tea plantations, improve tea quality, and enhance yields.

In addition to the benefits and contributions highlighted in the conclusion, this study also has certain limitations that need to be acknowledged. Firstly, the use of a limited dataset may not fully capture the diversity of tea diseases. Including a wider range of tea diseases would enhance the representativeness and applicability of the detection system. Secondly, biases in the training data, such as imbalances in disease instances or variations introduced by different image acquisition systems, could affect the performance of the tea disease detection system. Efforts should be made to address these biases and enhance the system’s robustness. Additionally, the study focuses on offline detection, which may not be practical for real-time implementation in tea plantations. Future research should explore real-time implementation, taking into account the resource and time constraints associated with practical deployment. Lastly, interpreting the decision-making processes of the deep learning model is challenging due to their complexity. Enhancing the interpretability of the model would enhance its usefulness in decision-making for tea farmers. Addressing these limitations can improve the practicality and effectiveness of tea disease detection systems.

## Data availability statement

The original contributions presented in the study are included in the article/supplementary material. Further inquiries can be directed to the corresponding author. The raw data can be accessed at the following link: https://www.jianguoyun.com/p/DRwyMxYQqJnmCxiGl5IFIAA.

## Author contributions

YS: Writing – original draft, Supervision. FW: Methodology, Data curation, Writing – review & editing. HG: Investigation, Funding acquisition, Writing – review & editing. RL: Writing – review & editing, Conceptualization. JY: Writing – review & editing, Resources, Visualization. JS: Writing – review & editing, Software.
